# Obituary

**Published:** 1893-10

**Authors:** 


### Obituary.

Dr. Walter Vought, of New York City, died at the New York Hospital, on Sunday, September 24, 1893, of typhoid fever, contracted, it is alleged, while attending a child ill of that disease. Dr. Vought was thirty-one years of age, a promising young physician, and was the son of Annie M. and the late John H. Vought of this city. His remains were brought to Buffalo for interment.

Dr. Graily Hewitt, of London, one of the best known medical men of his time, is dead at the age of sixty-five years. He was professor of obstetric medicine in the university, and obstetric physician to University Hospital, London. He was a voluminous writer, a teacher of great fame, and, altogether, one of the most genial and lovable characters that Great Britain has lately produced in the field of medicine. His loss will be seriously felt not only in the immediate locality of his work, but throughout all the world of obstetric and gynecological medicine.

Dr. Charles L. Dayton died at his residence, No. 400 Dearborn street, Buffalo, N. Y., on Thursday, September 7, 1893, in the sixty-seventh year of his age. His fatal ailment was typhoid fever, and had been of about ten days' duration.

Dr. Dayton was born in Eden, Erie county, N. Y., and was graduated in medicine from the University of Buffalo in 1854. Immediately thereafter he began the practice of his profession at Black Rock, and had been an active practitioner of medicine for nearly forty years, when he died. His family practice was large, and he was a man of genial companionship and of a sympathetic nature. He had been Health Physician of Buffalo, which was the only public office that he ever held. He leaves a wife and one daughter, besides his brother, Dr. Louis P. Dayton, who was formerly Mayor of Buffalo.

The funeral was largely attended, on Saturday, September 9th, and was conducted with Masonic rites.

He was a member of the Medical Society of the County of Erie, that has taken action relating to his death as follows :

At a special meeting of the Medical Society of the County of Erie, held September 8th, for the purpose of taking suitable action upon the death of Dr. Charles L. Dayton, the President, Dr. John Parmenter, opened the meeting in appropriate terms, then called for remarks by members of the profession.

The older members present who were well acquainted with Dr. Dayton, readily responded. Those who addressed the meeting in eulogy of the deceased, were Dr. Conrad Diehl, Dr. Hauenstein, Dr. Phelps, and Dr. Samo. The following resolutions were reported and read :

Whereas, This society has sustained a great loss in the death of Dr. Charles L. Dayton, one of its oldest members ; therefore,

*Resolved*, That while we bow to this dispensation of Divine Providence, we desire to place on record our appreciation of the many virtues of the deceased ; his never-failing devotion to his profession, and his high ethical bearing towards his brother practitioners, endeared him to all. His was a spirit of the true philanthropist, making him a faithful friend to the poor, a sympathetic confidant and adviser to the sick and suffering, and a public-spirited citizen, faithful and efficient in the discharge of official duties as Health Officer in times of great peril to the community.

*Resolved*, That these resolutions be spread upon the minutes of the Society, and a copy be transmitted to the family of our deceased brother.

*Resolved*, That the members of this society attend the funeral in a body.

WM. C. PHELPS,

JNO. J. WALSH,

GEO. F. COTT,

*Committee.*

GEO. F. COTT,

*Asst. Secretary.*

